# Outcomes of living-donor liver transplantation for Budd–Chiari syndrome: a retrospective comparative cohort study

**DOI:** 10.1097/MS9.0000000000005731

**Published:** 2026-10-19

**Authors:** Hafiz Aamir Bashir, Kaleem Ullah, Shams Uddin, Umar Baloch, Hafiz Syed Usman Ali Rizvi, Hafiz Bilal Ahmad, Abdul Wahab Dogar, Sidhant Ochani, Asaduzzaman Nur

**Affiliations:** a Department of Liver Transplantation, Pir Abdul Qadir Shah Jeelani Institute of Medical Sciences, Gambat, Pakistan; b College of Public Health, Ziauddin Medical College, Karachi, Pakistan; c Faculty of HPB Surgery, Enam Medical College, Dhaka, Bangladesh

**Keywords:** Budd–Chiari syndrome, caval replacement, liver transplantation, living donor, outcome

## Abstract

**Introduction:**

Living-donor liver transplantation (LDLT) for Budd–Chiari syndrome (BCS) poses unique technical and management challenges. The outcomes of LDLT for BCS, compared with other indications, are unclear and warrant further elucidation.

**Methods:**

Data from 26 BCS recipients (20 adults and 6 children) who underwent LDLT between January 2019 and June 2023 were retrospectively analyzed to evaluate outcomes. In addition, adult BCS recipients (*n* = 20) were compared with a matched control group of recipients (*n* = 50) transplanted for other indications for perioperative parameters and outcomes.

**Results:**

The median age of adult BCS recipients was 25 years, while that of pediatric patients were 11 years. The majority (84.62%) of BCS recipients were male, and all (100%) had a primary form of the disease. The most common symptom was ascites (73.07%). Only five patients (19.23%) underwent radiological intervention before transplantation. Retrohepatic caval replacement was performed in five patients (19.23%). Clavien–Dindo major morbidity (grade 3b/4) was documented in eight patients (30.76%). Hepatic arterial thrombosis and venous outflow obstruction were documented in three patients (11.53%) each. Compared with the control group, BCS recipients experienced prolonged cold ischemia and operative time, heightened blood loss, a greater need for transfusions, a longer hospital stay, and a higher incidence of outflow obstruction (*P* < 0.05). The BCS cohort had 1-year and 3-year survival rates of 84.6 and 73.1%, respectively, compared with 90 and 82% in the non-BCS group (log-rank, *P* = 1.08).

**Conclusion:**

Although patients with BCS present technical challenges, they can achieve outcomes comparable to those of non-BCS recipients with a good operative strategy, optimal postoperative anticoagulation, and regular surveillance for outflow complications.

## Introduction

Budd–Chiari syndrome (BCS) is a rare liver disease with a multifactorial etiology. BCS is not a primary disease of the liver parenchyma but occurs due to hepatic venous outflow obstruction at the level of the hepatic venules, hepatic veins (HVs), and inferior vena cava (IVC)[1]. The disease is more common in young adulthood, with an incidence of 0.2–2 cases per one million[2]. The primary form of BCS occurs due to outflow thrombosis or the presence of internal membranes or webs in the HVs or the IVC. In the secondary form of BCS, hepatic venous outflow obstruction is caused by compressive effects, such as tumors pressing on the HVs or the IVC[3].

HIGHLIGHTS

- The study analyzed 26 Budd–Chiari syndrome (BCS) recipients who underwent living-donor liver transplantation, with a male predominance (84.62%) and a median age of 25 years among adults. The 1- and 3-year survival rates for BCS recipients were 84.6 and 73.1%, respectively.

In BCS, persistent hepatic venous outflow obstruction results in increased sinusoidal pressure and hepatic congestion, which cause gradual centrilobular fibrosis with nodular regenerative hyperplasia, leading to cirrhosis, portal hypertension, and liver failure^[^4,5^]^. The clinical presentation can be acute, subacute, or chronic. The chronic form, which is much more common, manifests with features of gradual liver failure[6].

The EASL and AASLD practice guidelines recommend anticoagulation as the first-line treatment, while angioplasty/stenting is recommended for short-length stenosis[7]. Trans jugular intrahepatic portosystemic shunt (TIPS) is the next step, while liver transplantation (LT) is the last treatment option[8]. LT becomes mandatory in approximately 10–20% of BCS patients when other options are not appropriate[9].

LT for patients with BCS poses several challenges, such as technical difficulties in dealing with a congested, hypertrophied liver, extensive collateral formations, and an often-distorted vena cava, which can lead to massive blood loss. Similarly, postoperative management can make it difficult to maintain a balance between hemostasis and therapeutic anticoagulation. Moreover, in the long term, there may be problems with outflow obstruction and disease recurrence[10]. The outcomes of deceased-donor liver transplantation (DDLT) for BCS have been reported, with 5-year survival rates ranging from 65 to 95%^[^11–13^]^.

Living donor liver transplantation (LDLT) is more technically challenging than DDLT. LDLT for patients with BCS has the additional limitation of the unavailability of a donor vena cava for replacement of a thrombosed recipient vena cava. Moreover, data on the outcomes of LDLT in BCS are limited. Previously reported data are in the form of case series^[^14,15^]^.

This study aimed to report the outcomes of BCS recipients who underwent LDLT. This study comprised a large single-center series of BCS patients who had undergone LDLT. We compared the demographics, operative parameters, and postoperative complications of BCS recipients with a matched non-BCS LDLT cohort. Reporting outcomes of LDLT for BCS is essential for gaining a comprehensive understanding of the multifaceted experiences and challenges faced by liver recipients and transplant professionals. This cohort study has been reported in line with the STROCSS and TITAN 2025 guidelines^[^16,17^]^.

## Materials and methods

### Study setting and design

This is a retrospective comparative cohort study which was conducted at the liver transplant department of a tertiary care hospital in Pakistan, following the principles of the Declaration of Helsinki (2008) and “good clinical practice” guidelines, after approval by the institutional ethics committee. We reviewed a prospectively collected database of all adults and children with BCS (*n* = 26) who underwent LDLT between January 2019 and June 2023. It was supplemented with inpatient and outpatient clinical records, along with subsidiary databases of radiological and endoscopic interventions.

### Patient selection and inclusion criteria

#### Inclusion

All BCS patients had a confirmed diagnosis of BCS based on clinical presentation and imaging evidence of hepatic venous outflow obstruction.

### LT candidacy

Patients with confirmed BCS were evaluated for LT if they met any of the following criteria: progressive liver failure or decompensated liver disease; failure of non-transplant interventions (e.g., shunts and angioplasty); presence of hepatocellular carcinoma that met the University of California, San Francisco criteria. All eligible LDLT BCS patients within the study period were included.

### Exclusion criteria

Patients undergoing re-transplantation and those with incomplete clinical data were excluded from the study.

### Diagnostic and etiological evaluation of BCS

These patients were classified as acute, subacute, or chronic based on their clinical presentation. All patients underwent a workup for inherited and acquired thrombophilias. If necessary, a bone marrow biopsy was performed to rule out any underlying prothrombotic disorders. Those with no known etiology after evaluation were classified as idiopathic. To evaluate the HVs, IVC patency, the porto-mesenteric venous system, and prior stents in detail, these patients underwent triphasic contrast-enhanced abdominal computed tomography (CT) with an additional 3-minute delayed phase.

Donor and recipient evaluation processes have been thoroughly explained elsewhere. All potential donors were screened for procoagulant tendencies[18].

### Recipient operative details

Tense, congested livers in BCS patients presented significant challenges during surgery. Initial liver mobilization and splitting of the triangular ligament were particularly difficult. To mitigate this, we employed a “porta-first” dissection technique. Proximal hepatic arterial control was achieved with vascular bulldog clamping, after which high hilar arterial ligation was performed to reduce liver congestion before hepatectomy. We also maintained a low threshold for early division of the porta hepatis and portocaval shunting to minimize the anticipated heavy blood loss during hepatectomy.

Liver mobilization from the IVC required careful dissection due to dense pericaval fibrosis, adhesions between the diaphragm and liver, extensive collateral formation, and marked hypertrophy of the caudate lobe. All these factors made explant hepatectomy extremely difficult. These challenges were addressed with careful dissection and ligation of all collateral veins.

After explant hepatectomy, the fibrous tissues surrounding the IVC were carefully dissected. The dissection was extended cranially to the suprahepatic IVC up to the diaphragmatic crus. Similarly, the dissection was extended caudally until the suprarenal IVC was reached. After completing the dissection, supra- and infra-IVC control was secured with the application of vascular cross-clamps. It is noteworthy that the veno-venous bypass procedure was not employed in any patient. The IVC was then opened at the site of the hepatic venous orifices, and its lumen was inspected specifically for webs, bands, and thrombosis. If any web or band was found, it was excised, and the lumen was cleared to ensure a patent cava for implantation. This was facilitated by extended IVC mobilization and the use of total caval clamping techniques. Additionally, a controlled digital assessment of the cavo atrial junction was performed to rule out stenosis. The cavo-atrial junction was approached transabdominally via the diaphragm.

Preferably, the native recipient vena cava was preserved if it was judged to be healthier for implantation. For right lobe graft implantation, the margins of the right HV’s orifice, and for left lobe grafts, both the left and middle HVs’ orifices, along with part of the IVC margins, were excised and refreshed. The excision of the thickened vein wall was extended both cranially and caudally until a healthier caval wall was reached. This technique of resection of the HV margins with cavoplasty facilitated direct anastomosis with the graft veins on the native IVC via a large orifice. Right lobe grafts required extension of all HVs ≥5 mm in diameter (segment 5/8 veins) toward the right HV orifice using synthetic vascular grafts to form a common anastomosis site for a single anastomosis. For the graft inferior HV anastomosis, a separate orifice on the IVC was created for anastomosis.

The vascular anastomosis was performed using the eversion technique with a 5-0 polypropylene suture in a continuous fashion, while the other stages of the implantation followed standard procedures. In the majority of cases in which native cava was preserved, the graft implantation was completed with the cava cross-clamped, except in a single case in which the IVC was selectively side-clamped due to cardiac compromise.

### Surgical techniques for thrombosed/stenosed IVC in BCS recipients

The retrohepatic IVC excision and reconstruction were performed with a synthetic graft if the IVC was found to be chronically thrombosed or stenotic, or if previous stenting procedures had thinned its wall. A polytetrafluoroethylene (PTFE) graft of adequate size (2 cm) and with a length equal to that of the thrombosed segment was used for IVC replacement.

### Postoperative management

In the early postoperative period, all patients who underwent BCS received anticoagulation with low-molecular-weight heparin (enoxaparin) at a therapeutic dose of 1–2 mg/kg, which was transitioned to oral rivaroxaban at hospital discharge. All patients concurrently received antiplatelet therapy, and both drugs were continued for the rest of their lives.

Patients underwent daily Doppler scans to check vascular patency for the first 5 days and thereafter as needed until discharge. Surveillance Doppler ultrasonography was continued during follow-up to determine vascular patency. Standard immunosuppression consisted of tacrolimus and corticosteroids. Mycophenolate mofetil was added selectively for patients considered to be at higher risk of rejection. Prednisolone was gradually tapered over a 3-month period. Tacrolimus trough levels were targeted to 6–9 ng/mL during the first 6 months and adjusted to 4–6 ng/mL thereafter.

A scheduled Doppler ultrasound monitoring protocol was implemented. Studies were performed fortnightly during the first month, followed by assessments at 3, 6, and 12 months. Subsequently, monitoring continued a semi-annual (6-monthly) basis. Any abnormalities in flow and velocity metrics detected on Doppler ultrasonography raised suspicion of vascular complications, necessitating confirmatory CT angiography.

## Definitions

Postoperative acute kidney injury (AKI) was defined as serum creatinine >1.5 mg/dL or an increase >0.3 mg/dL within 48 hours, or urine volume <0.5 mL/kg/h for more than 6 hours. Early allograft dysfunction was defined as bilirubin ≥10 mg/dL on day 7, INR ≥1.6 on day 7, and alanine or aspartate aminotransferase >2000 IU/L within the first 7 days. Early venous outflow obstruction was defined as occurring within 90 days, and late obstruction as occurring beyond 90 days.

Prolonged ascitic drainage was defined as >1000 mL of ascitic drainage 14 days after the index surgery[14].

## Matched cohorts

A comparative analysis was performed between two cohorts of adult LDLT recipients: 20 patients with BCS (BCS cohort, *n* = 20) and 50 patients in a control group of non-BCS recipients (non-BCS cohort, *n* = 50). The control group was selected via random sampling using a 1:3 matching ratio (BCS: non-BCS). Matching criteria ensured comparability across key demographic and clinical variables, specifically recipient age, body mass index (BMI), model for end-stage liver disease (MELD) score, graft type, and graft-to-recipient weight ratio (GRWR). A CONSORT-style flow diagram (Supplemental Digital Content Figures S1, available at: https://links.lww.com/MS9/B396) outlines case screening, exclusions, and final cohort selection. Supplemental Digital Content Figures S1–S8, available at: https://links.lww.com/MS9/B396.

## Statistical analysis

Data regarding patient demographics, clinical presentation, disease severity, perioperative events, postoperative course, and early- and long-term survival were analyzed using our prospective database. Descriptive methods were used to present the data for the entire BCS cohort. Data are presented as mean (standard deviation) or median (interquartile range) for continuous variables and as number (percentage) for discrete variables. The perioperative outcomes and survival rates of the adult BCS cohort were compared with those of a matched non-BCS adult LDLT cohort (1:3). The BCS and control groups were compared using standard statistical tests. The unpaired Student’s *t*-test or Mann-Whitney *U* test was used to compare continuous variables, while the chi-square test or Fisher’s exact test was used to compare discrete variables between the BCS and control cohorts. Kaplan–Meier curves were used to estimate actuarial survival rates, and the log-rank test was used to compare survival between the two groups. Statistical significance was set at *P* < 0.05. SPSS version 25 was used for the statistical analysis.

## Results

During the study period, 863 liver transplants were performed, and all patients underwent LDLT. No patients receiving deceased donor transplants were included in the study. ABO-compatible LDLT was performed in all BCS patients. Among these, 26 patients – 20 adults and 6 children – underwent LDLT for BCS. The demographics, clinical features, and preoperative characteristics of all BCS patients are presented in Table 1.

**Table 1:** Demographics of LDLT-BCS patients.

Parameters	*n* = 26
Age in years (median)	
Adult (*n* = 20)	25.0 (1948)
Children (*n* = 6)	11.0 (516)
Gender	
Male	22 (84.62%)
Female	4 (15.38%)
Etiology	
Primary BCS	26 (100%)
Myeloproliferative diseases – JAK 2 mutation	2 (7.69%)
Anti-phospholipid antibody syndrome	2 (7.69%)
Protein C and S, AT 3 deficiency	18 (69.23%)
IVC webs	7 (26.92%)
Idiopathic	5 (19.23%)
Secondary BCS	0
Mode of presentation	
Sub-acute	1 (3.84%)
Chronic	25 (96.15%)
Clinical presentation	
Ascites	19 (73.07%)
Jaundice	18 (69.23%)
Hepatic encephalopathy	4 (15.38%)
Spontaneous bacterial peritonitis	3 (11.53%)
Bloodstream infection (BSI)	6 (23.07%)
Acute kidney injury	3 (11.53%)
Site of hepatic venous outflow block	
Hepatic veins (HV) alone	20 (76.92%)
Inferior vena cava (IVC) alone	0
IVC thrombosis along HV obstruction	6 (23.07%)
Child-Pugh score	
Child B	16 (61.53%)
Child C	10 (38.46%)
MELD score at transplant (median)	17.50 (11–24)
Prior radiological vascular interventions	
Angioplasty/stenting	5 (19.23%)
Portal vein thrombosis	3 (11.53%)
Yerdel’s type 1/2	0
Yerdel’s type 3	2 (7.69%)
Yerdel’s type 4	1 (3.84%)
Indication for transplant	
Progressive liver dysfunction	26 (100%)
Hepatocellular carcinoma	0

### Perioperative details of the BCS cohort

#### Adults’ recipients (*n* = 20)

All patients (100%) had primary BCS with an underlying procoagulant condition. Ascites and jaundice were the most common symptoms. All patients had Child–Pugh class B or C cirrhosis. Five patients (25%) underwent percutaneous decompression before LT. Preoperative portal vein thrombosis (PVT) was identified in two adult patients (10%). Both had Yerdel grade III thrombosis and underwent successful eversion thrombectomy. The native cava was preserved in 15 patients (75%), while IVC replacement using a PTFE graft was required in 05 (25%). Luminal web excision was needed in seven patients (35%). Standard arterial and duct-to-duct biliary reconstruction was performed in all adult patients with BCS.

#### Pediatric recipients (*n* = 06)

The median age of the pediatric BCS LDLT recipients was 11 years (5–16 years). All patients had primary BCS. Five children (83%) received a right-lobe graft, and a single child received a left-lobe graft. All patients underwent standard implantation with preservation of the native cava. A mesenterico-portal jump graft was required in one patient for grade IV PVT. Duct-to-duct biliary reconstruction was performed in five patients (83%), and Roux-en-*Y* reconstruction was performed in a single child.

### Early postoperative period morbidity and mortality of all BCS cohort (n = 26)

Major morbidity (Clavien-Dindo grade 3b/4) was documented in eight (30.76%) patients. AKI and bloodstream infections (BSI) were observed in six patients (23.07%) each, and two (7.7%) patients had early bile leaks. Re-exploration was required in four (15.38%) patients. One adult patient was re-explored for hepatic artery thrombosis (HAT) and PVT on post-operative day 7; the second was re-explored for PVT on post-operative day 15, and the third adult was re-explored for HAT on postoperative day 2. In addition, one child required re-exploration for HAT in the early postoperative period. Four patients (15.38%) required readmission within 90 days.

The 90-day mortality rate in the BCS cohort was 15.38% (4/26). One patient developed graft dysfunction and sepsis after two re-explorations for HAT and PVT and eventually died. Another patient developed AKI and BSI, progressed to multi-organ dysfunction syndrome, and died. The third patient also developed graft dysfunction and sepsis, leading to death on postoperative day 12. There was one case of pediatric mortality. The patient with a porto-mesenteric jump graft was re-explored for portal thrombotic episodes and eventually died secondary to multi-organ dysfunction.

### Late morbidity and mortality of all BCS cohort (n = 26)

The median follow-up period was 30 months (range: 0–51 months). No patients were lost to follow-up during the study period. Anastomotic biliary strictures developed in 05 patients (19.23%). Three patients (11.53%) developed late hepatic venous outflow obstruction. One patient developed thrombosis due to discontinuation of anticoagulants. His symptoms resolved once anticoagulant therapy was resumed. The other two patients were treated with venoplasty by an interventional radiologist. Of the three patients with delayed venous outflow obstruction, two had a native cava, while one had replacement of the cava with a PTFE graft. Similarly, of the five patients who underwent preoperative radiological intervention, only one presented with late outflow obstruction. These relatively small numbers in various subgroups limit the ability to definitively assess the relationship between the anastomotic method and late venous outflow obstruction.

The 1- and 3-year survival rates among patients with BCS were 84.6 and 73.1%, respectively. Three recipients (11.53%) died in the late follow-up period. One immunosuppressant-noncompliant recipient developed chronic rejection at 16 months. The second patient died of renal failure and sepsis at 24 months of follow-up. The third patient died of disseminated intravascular coagulation secondary to a myeloproliferative disorder at 19 months post-LDLT in Table 2.

**Table 2:** Intraoperative and postoperative details of all LDLT-BCS patients.

Parameters	Frequency
Graft type	
Right lobe	24 (92.30%)
Left lobe (one adult and one child)	2 (7.69%)
Left lateral segment	0
IVC status	
Inferior vena cava replacement	5 (19.23%)
Inferior vena cava preserved	21 (80.76%)
Portal vein thrombosis management (*n* = 2)	
Eversion thrombectomy	2 (7.69%)
Mesenterico-portal jump graft	1 (3.84%)
Biliary reconstruction	
Adults	*N* = 20
Duct to duct	20 (100%)
Roux-en-*Y* hepaticojejunostomy	0
Children	*N* = 6
Duct to duct	5 (83.33%)
Roux-en-*Y* hepaticojejunostomy	1 (16.66%)
Postoperative morbidity	
Early venous outflow obstruction (within first 3 months)	1 (3.84%)
Hepatic arterial thrombosis	3 (11.53%)
Portal vein thrombosis	4 (15.38%)
Bleeding	0
Biliary leak	2 (7.69%)
Bloodstream infection (BSI)	6 (23.07%)
Acute kidney injury (AKI)	6 (23.07%)
Prolonged ascitic drainage	2 (7.69%)
Reoperation	4 (15.38%)
Readmission	4 (15.38%)
Major morbidity (Calvien–Dindo grade 3b/4)	8 (30.76%)
90-day mortality	4 (15.38%)
Median ICU stay (in days)	7.5 (5.21)
Median hospital stays (in days)	15 (9.27)
Median follow-up (months)	30 (0.51)
Biliary strictures	5 (19.23%)
Late venous outflow complications (after 3 months)	3 (11.53%)
Survival	
1-year survival	22 (84.61%)
3-year survival	19 (73.07%)

### Comparison of adult BCS (n = 20) with the matched non-BCS cohort (n = 50)

The demographic, preoperative, intraoperative, and postoperative parameters of adult LDLT recipients (20 BCS and 50 non-BCS) were compared (Table 3). This comparison showed a statistically significant difference between the BCS and non-BCS cohorts in terms of cold ischemia time (*P* = 0.005), anhepatic phase (*P* = 0.001), blood loss (*P* = 0.007), and blood transfusions (*P* = 0.014). The need for reoperation, hospital readmission, and 90-day mortality were not significantly different between cohorts. Moreover, the BCS cohort had an increased length of hospital stay (*P* = 0.002) and higher rates of late venous outflow complications (*P* = 0.021). One- and 3-year survival rates of the BCS and matched non-BCS cohorts were 85% vs. 90 and 70% vs. 82%, respectively (log-rank, *P* = 0.108) (Table 3)

**Table 3:** Comparison of BCS adult LDLT patients and non-BCS LDLT patients.

Preoperative factors	Cases (*n* = 20)	Controls (*n* = 50)	*P*-value
Mean age (years)	27.50 (±8.43)	28.68 (±1.82)	0.348
Gender (male)	17 (85%)	41 (82%)	1.000
Weight (kg)	64.05 (±5.54)	62.56 (±1.93)	0.099
Mean BMI kg/m^2^	22.35 (±2.05)	21.56 (±1.23)	0.052
Mean MELD score	18.05 (±5.12)	18.86 (±4.96)	0.543
Ascites	19 (95%)	33 (66%)	**0.014**
Preoperative AKI	3 (15%)	5 (10%)	0.680
PVT	3 (15%)	2 (4%)	0.137
Donor and graft factors			
Donor age	25.15 (±3.49)	24.78 (±4.87)	0.758
Mean GRWR	1.11 (±0.20)	1.13 (±0.19)	0.664
Graft type (right lobe)	19 (95%)	50 (100%)	0.999
Intra-operative factors			
Mean cold ischemia time CIT (min)	13.50 (±1.60)	12.24 (±1.64)	**0.005**
Mean anhepatic time (min)	114.25 (±15.06)	72.28 (±11.12)	**0.001**
Mean intraoperative blood loss (mL)	1680 (±294)	1356 (±484)	0.007
Mean packed RBC transfusion	2.0 (±0.72)	1.2 (±1.34)	**0.014**
Total blood product transfusion	3.15 (±1.26)	1.66 (±1.97)	**0.003**
Mean duration of surgery (min)	580 (±70)	531 (±73)	**0.004**
Postoperative morbidity and outcomes			
Acute kidney injury	6 (30%)	4 (8%)	**0.027**
Bloodstream infections	5 (25%)	4 (8%)	0.055
Bleeding	0	2 (4%)	1.000
Early allograft dysfunction	3 (15%)	2 (4%)	0.137
Hepatic arterial thrombosis	3 (15%)	3 (6%)	0.343
Early outflow obstruction (within 3 months)	1 (5%)	0	0.286
Biliary leak	6 (30%)	4 (8%)	**0.017**
Re-exploration	3 (15%)	2 (4%)	0.137
Acute cellular rejection	0	3 (6%)	0.552
Median ICU stay (days)	9 (521)	7 (512)	0.009
Median hospital stay (days)	15 (927)	13 (827)	**0.002**
Hospital readmission	4 (20%)	7 (14%)	0.533
Late outflow obstruction (after 03 months)	3 (15%)	0	**0.021**
Major morbidity (Calvien–Dindo grades 3b/4)	7 (35%)	10 (20%)	0.006
90-days mortality	4 (20%)	2 (4%)	0.051
Overall survival	14 (70%)	41 (82%)	0.269

Values in bold are statistically significant (*p* < 0.05).

## Discussion

Various modalities are used to treat patients with BCS. All these modalities aim to minimize outflow obstruction. LT remains the definitive treatment when other modalities fail. Approximately 10–20% of patients with BCS undergo LT. Studies from Western countries have reported good outcomes of DDLT for BCS. DDLT has the advantage of replacing the recipient’s stenosed/fibrotic retrohepatic IVC with the donor IVC, which may be why DDLT has been recognized as a successful treatment option for BCS, as evidenced by favorable long-term outcomes demonstrated by numerous studies and registry databases^[^11,19^]^.

In contrast, all studies related to the BCS outcome have been published by Asian centers, where the organ pool is largely based on living donors. There are limitations in utilizing the donor cava in LDLT settings for BCS patients with a deformed cava. However, synthetic grafts or cryopreserved vessels can be used for this purpose. The LDLT experience for BCS is limited, consisting mainly of single-center series with a limited number of cases (Table 4). This low number is most likely explained by well-recognized technical difficulties. Furthermore, a substantial number of BCS patients present with caval thrombosis, which necessitates caval replacement for a successful LDLT outcome.

**Table 4:** Studies published on LDLT on BCS patients.

Series	(*N*)	Morbidity (%)	Mortality (%)	Mean follow-up (months)	1-year survival (%)	3-year survival (%)
Our study (2024)	26	30.76	15.38	30	84.61	73.07
Gunasekaran *et al* (2020)[19]	24	37.5	12.5	28.6	83.3	71
Young-In yoon *et al* (2019)[20]	5	-	Nil	126	1	100
Fatih Gonultas *et al* (2019)[21]	17	35.2	Nil	-	-	-
Umeshita *et al* (2019)[18]	57	-	-	-	89.5	83.7
C. Karaca *et al* (2017)[15]	22	31.8	18.2	43	-	-
Pahari H *et al* (2016)[22]	9	44.4	11.1	15.7	-	-
Cengiz Ara *et al* (2016)[14]	39	48.7	33.3	16.1	-	-
Dogrul AB *et al* (2015)[23]	10	-	20	10.5	-	-
Bas *et al* (2012)[24]	3	33.3	Nil	18	-	-
G.S Choi *et al* (2010)[25]	4	-	Nil	19.5	-	-
Yamada T *et al* (2006)[17]	9	-	11.1	-	-	-

In this study, we report the demographics, clinical presentations, operative parameters, and outcomes of one of the largest series of BCS recipients (*n* = 26; 20 adults and 6 children) who underwent LDLT. It is important to note that this study is descriptive and exploratory, and it does not allow for formal equivalence testing of the observed outcomes.

Umeshita *et al*[26] reported the largest BCS cohort of 57 patients who underwent LDLT. However, they reported only survival rates without providing detailed peri-operative parameters for these patients.

Additionally, Ara *et al*[14] reported a series of 39 BCS patients who underwent LDLT. They reported detailed peri-operative parameters along with morbidity and mortality rates. However, they did not report survival rates for their BCS cohort.

A recent study by Gunasekaran *et al*[27] from a neighboring country also reported one of the largest BCS cohorts (*n* = 24) undergoing LDLT. They reported the demographics, operative parameters, morbidity, and survival of these patients.

A local study from Pakistan, conducted by Parkash *et al*[28] in 2017, reported the characteristics and treatment offered to 45 patients with BCS. The main treatment provided to the patients was anticoagulation or TIPS. None of the patients underwent LDLT owing to the unavailability of a transplant program, whereas all patients reported in this study underwent LDLT.

In our cohort, most transplant patients were male (84.6%). The local study by Parkash *et al*[28] also reported male predominance in their cohort. This may be due to Pakistan’s patriarchal society. Similarly, a study conducted by Gunasekaran *et al*[27] in India also reported male predominance among patients with BCS who underwent LT. However, another Indian study reported that the prevalence of BCS was higher in females between the ages of 31 and 40 years[29].

All our patients had primary BCS. Protein C and S deficiencies were observed in 69.2% of patients. IVC webs were found in seven patients (26.92%), while 19.23% were labeled idiopathic. A local study conducted by Parkash *et al*[28] identified protein C and S deficiencies in 78.6 and 60.7% of patients, respectively. The majority of BCS patients (90%) reported by Gunasekaran *et al*[27] were classified as primary, and 10% as secondary. They found caval webs only in 11.11% of patients compared to 26.92% in our cohort. Owing to the high prevalence of caval webs in our BCS cohort, we routinely inspected for caval webs irrespective of IVC morphology in all our BCS patients. Membrane obstruction of the IVC has also been reported in BCS patients in other Eastern studies^[^11,20^]^. It is crucial to excise these webs for good outflow. In one of our patients, the caval segment became thinned and deformed while excising the webs. The thinned-out segment was removed, and end-to-end anastomosis of the caval segment was performed.

Only 19.23% of our patients underwent radiological intervention before transplantation. This low number suggests limited availability of interventional radiology services for these patients in Pakistan. We observed preoperative AKI and SBP in three patients (11.5%) and BSI in six patients (23.1%). BCS patients usually require regular paracentesis, which predisposes them to AKI and infections^[^21,27^]^. The study conducted by Gunasekaran *et al*[27] also reported similar findings, i.e., a 21% incidence of AKI, SBP, and BSI.

In our cohort, 19.23% of patients had combined hepatic venous and caval thrombosis. Similar rates were reported by others: 20% by Gunasekaran *et al*[27] and 18% by Ara *et al*[14]. This is a significant proportion in the LDLT setting.

In comparing BCS with non-BCS cohorts, we recorded a longer cold ischemia time in the BCS group, and the reason for this was the multiple venous reconstructions in the BCS group. The anhepatic time in BCS patients was also longer than in the non-BCS group, and the reason for this was routine IVC mobilization, cross-clamping, caval lumen inspection, and venoplasty, and, in a few cases, caval resection and replacement with a graft. Greater operative blood loss was observed in the BCS group than in the non-BCS group, and this greater blood loss was attributable to the complexity of recipient surgery in the BCS group and probably to the prolonged anhepatic phase in these patients. Consequently, BCS recipients received blood product transfusions more frequently than the non-BCS cohort. Other centers have also reported greater operative blood loss and more frequent blood transfusions in BCS recipients^[^22,27^]^.

Similarly, our operative time for BCS recipients was significantly longer. Hepatectomy remains challenging in BCS patients, as congested livers do not provide a working space, and dense adhesions and extensive collateral vessels make the task challenging.

In LDLT, the approach to venous reconstruction needs to be tailored primarily to the imaging and operative findings. Our center is primarily LDLT-based, and we used only synthetic grafts to replace the thrombosed vena cava because of a lack of cryopreserved vascular grafts.

We routinely performed caval mobilization. This approach not only facilitated detailed inspection of the deformed cava but also, at times, helped restore continuity without the need for synthetic grafts when up to 3 cm of deformed or thinned-out caval segments required excision. However, caval replacement with a synthetic graft was still needed in 19.3% of our BCS patients. On follow-up imaging, four patients had patent PTFE grafts, while one patient developed partial thrombosis. Yoon *et al*[22] also reported the use of a synthetic graft for caval reconstruction in five patients. Ara *et al*[14] also reported caval replacement in 15.38% of patients; however, they used cryopreserved aortic grafts for caval replacement. Both series showed good long-term patency similar to our observations; however, Yoon *et al*[22] reported one patient with a redundant, folded synthetic graft on CT imaging.

Post-transplant delayed venous outflow anastomotic narrowing represents a substantial source of morbidity in BCS recipients[22]. In our study, late venous outflow obstruction was experienced in 15% of patients. In one patient, thrombosis at the venous anastomotic site occurred due to discontinuation of anticoagulation. However, the thrombus resolved once the anticoagulation was resumed. The other two patients with outflow narrowing were treated with serial dilatation by an interventional radiologist. We started anticoagulation in BCS recipients with enoxaparin post-LDLT. Enoxaparin was then switched to oral rivaroxaban at discharge. Similar to some other coumarin derivatives, rivaroxaban does not require regular monitoring. However, the long-term anticoagulation effect of oral rivaroxaban in these patients cannot be commented upon and warrants further investigation. Recurrent venous outflow obstruction in BCS recipients was also reported as a significant morbidity by other centers ^[^22,27^]^. Ara *et al*[14] also reported that two of 26 surviving post-LDLT BCS recipients developed outflow complications and were treated with interventional radiological methods. Similarly, Gunasekaran *et al*[27] also documented significantly higher outflow complications compared to non-BCS recipients post-LDLT. This outflow obstruction in BCS patients might be related to the surgical technique, or to performing an anastomosis on an unhealthy, fibrotic cava. The underlying hypercoagulability of these patients may also contribute to this morbidity. It is therefore important to address all these factors. Noncompliance can be a serious issue in BCS patients and can only be addressed with continuous patient education.

Moreover, BCS recipients require regular imaging surveillance in post-transplant settings for early detection of asymptomatic narrowing of venous outflow, as observed in one of our patients, in whom mild narrowing was noted at a 1-year post-transplant follow-up. The patient was managed with serial dilatation by an interventional radiologist. For that reason, we suggest that regular imaging surveillance might be beneficial for these recipients.

Outcomes of LDLT for BCS recipients are associated with a slightly increased mortality compared to other recipients, as evidenced by series published by multiple centers. The comparison of survival rates from other LDLT centers is presented in Table 4. No late mortality due to disease recurrence-related complications was observed in our series. However, the slight increase in early mortality may be attributed to the fragility of these patients.

The main limitation of this study was its retrospective design at a single LDLT center. We also lacked a detailed etiologic evaluation for some cases, which may be necessary to assess the long-term benefit of anticoagulation. Furthermore, the relatively small numbers in various subgroups limit the ability to definitively assess how anastomosis method and prior intervention before LT relate to late outflow obstruction. Future research with larger cohorts will be necessary to determine these associations. Despite these limitations, this study is one of the largest series reporting detailed LDLT outcomes for BCS recipients.

## Conclusion

LDLT is a suitable choice for individuals with symptomatic BCS who do not respond to other modalities. Patients with BCS are difficult to transplant in LDLT settings because they pose various challenges. However, they can have outcomes broadly comparable to non-BCS patients with good peri-operative strategies and postoperative anticoagulation. Our findings of comparable outcomes require validation in larger, adequately powered, multicenter studies.

## Data Availability

Not applicable.
